# Maternal Omega-6/Omega-3 Concentration Ratio During Pregnancy and Infant Neurodevelopment: The ECLIPSES Study

**DOI:** 10.3390/nu17010170

**Published:** 2025-01-02

**Authors:** Behnaz Shahabi, Carmen Hernández-Martínez, Cristina Jardí, Estefanía Aparicio, Victoria Arija

**Affiliations:** 1 Nutrition and Mental Health (NUTRISAM) Research Group, Universitat Rovira i Virgili, 43201 Reus, Spain; behnaz.shahabi@urv.cat (B.S.); carmen.hernandez@urv.cat (C.H.-M.); cristina.jardi@urv.cat (C.J.); estefania.aparicio@urv.cat (E.A.); 2 Institut d’Investigació Sanitària Pere Virgili (IISPV), 43003 Tarragona, Spain; 3 Research Center for Behavioural Assessment (CRAMC), Department of Psychology, Universitat Rovira i Virgili, 43007 Tarragona, Spain

**Keywords:** unsaturated fatty acids, omega-3 fatty acids, *n*-3, omega-6 fatty acids, *n*-6, blood chemical analysis, infant, neurodevelopment, pregnancy

## Abstract

Background: The balance of omega-6/omega-3 (*n*-6/*n*-3) is crucial for proper brain function as they have opposite physiological roles. Objectives: To analyze the association between maternal serum ratios of *n*-6/*n*-3 in the first and third trimesters of pregnancy and the neurodevelopment of their children in the early days after birth in the population of Northern Spain’s Mediterranean region. Methods: Longitudinal study in which 336 mother–child pairs participated. Mother serum concentrations of long-chain polyunsaturated fatty acids (LCPUFAs), docosahexaenoic acid (DHA), eicosapentaenoic acid (EPA), and arachidonic acid (ARA) were determined. Sociodemographic, clinical, lifestyle habits, and obstetrical variables were collected. The Bayley Scales of Infant and Toddler Development (BSID-III) was used to assess infant neurodevelopment. Multiple linear regression models adjusting for confounding factors were performed. Results: In the third trimester, a higher maternal *n*-6/*n*-3 ratio was negatively associated with infant motor development (β = −0.124, *p* = 0.023). Similarly, higher ARA/DHA ratios were negatively associated with total motor (β = −2.005, *p* = 0.002) and fine motor development (β = −0.389, *p* = 0.001). No significant associations were observed in the first trimester nor for the ARA/EPA ratio in the third trimester. Conclusions: Our findings indicate that an elevated *n*-6/*n*-3 ratio and ARA/DHA ratio in the third trimester of pregnancy are associated with poorer motor development outcomes in infants. These results highlight the importance of optimizing maternal fatty acid balance during pregnancy to support fetal neurodevelopment, suggesting a need for further research to verify these associations and elucidate underlying mechanisms.

## 1. Introduction

Prenatal brain development is greatly influenced by the nutritional intake received from the mother, highlighting the importance of maintaining an adequate nutritional status during pregnancy to promote healthy development [1]. Omega-6 (*n*-6) and omega-3 (*n*-3) long-chain polyunsaturated fatty acids (LCPUFAs) are a group of essential fatty acids with chains of 20 or more carbon atoms [2], which play a crucial role in various physiological processes, including brain development and function [3]. Among them, docosahexaenoic acid (DHA, *n*-3) and arachidonic acid (ARA, *n*-6) are particularly important for neurodevelopment being that these fatty acids are major components of neuronal membranes and are essential for key prenatal neurodevelopmental processes, including the formation and functioning of neuronal membranes, synapses, neurotransmitter release, and the regulation of overall brain function and development [3].

Previous research has primarily focused on the effects of *n*-3 LCPUFAs, particularly DHA, on infant neurodevelopment, with contradictory findings. For example, Rioux et al. [4] found no relationship between maternal DHA status at the end of pregnancy and infant neurodevelopment at 6 months. Similarly, Shahabi et al. reported that higher DHA serum concentrations during the first trimester were associated with poorer neurodevelopment during the first weeks of life [5]. In fact, the Cochrane Review of Randomized Controlled Trials (RCTs) and other systematic reviews indicate that current evidence is insufficient to conclusively demonstrate a positive impact of *n*-3 LCPUFA supplementation during pregnancy on offspring neurodevelopment [6,7]. A potential factor contributing to these inconclusive results may be the limited attention given to *n*-6 fatty acids and their interactions with *n*-3 fatty acids. Beyond their individual contributions, *n*-3 and *n*-6 LCPUFAs compete for incorporation into cell membranes [8] and exhibit opposing physiological roles. While *n*-6 fatty acids, particularly ARA, serve as precursors to pro-inflammatory eicosanoids such as prostaglandins and leukotrienes, *n*-3 fatty acids, such as EPA and DHA, are precursors to anti-inflammatory eicosanoids and resolvins that mitigate excessive inflammation [9]. For this reason, a dietary ratio of *n*-6/*n*-3 LCPUFA of 4:1 or less has been recommended [9], although a specific target for serum LCPUFA ratios remains undefined.

In the context of pregnancy and fetal neurodevelopment, the balance between these fatty acids is of particular importance due to the critical period of organism growth [10]. Maintaining an appropriate *n*-6/*n*-3 balance can promote optimal neurological development and reduce the risk of neurodevelopmental disorders associated with fatty acid imbalances [11]. However, studies evaluating the effect of maternal serum *n*-6/*n*-3 ratios during pregnancy on offspring neurodevelopment remain scarce and report contradictory results. For instance, in the Generation R study in the Netherlands, Steenweg-de Graaff et al. [12] found that higher *n*-6/*n*-3 ratios were associated with an increased risk of emotional symptoms in 6-year-old children. Similar findings were reported in the ABCD study and the INMA cohort in Spain, where higher *n*-6/*n*-3 ratios were linked to emotional, attentional, and hyperactivity problems in children aged 5–6 years [13,14]. In contrast, the study by Brouwer-Brolsma et al. [15], which assessed cognitive development in 7-year-old children, did not find any significant relationship. Several factors might contribute to these discrepancies, including differences in the timing, tissue type, and methods used to determine LCPUFA concentrations, as well as variations in the ages and neurodevelopmental outcomes assessed (e.g., cognitive development vs. psychopathological problems). For younger children, limited evidence is available. The Seychelles Child Development Study by Strain et al. [16] assessed the impact of maternal *n*-6/*n*-3 ratios during pregnancy on early cognitive outcomes. Their findings suggested an association between higher *n*-6/*n*-3 ratios and poorer psychomotor development at 9 months, measured using the Bayley Scales for Infant Development (BSID-II). However, this association was not significant at a follow-up conducted at 30 months, highlighting the need for further studies to clarify the role of *n*-6/*n*-3 balance in early neurodevelopment, particularly during the first months of life when the impact of postnatal environmental factors has not yet exerted a significant influence.

The timing of gestation in which environmental factors exert their influence on the developing brain is a critical consideration in prenatal research. Different neurodevelopmental milestones occur throughout pregnancy, such as neuronal proliferation, migration, and synaptogenesis, each of which may be differentially sensitive to specific factors depending on their timing. The first trimester is characterized by fundamental structural processes, including neurogenesis and neuronal migration, which lay the foundation for later brain development [17,18]. In contrast, the third trimester is marked by rapid fetal and brain growth, involving critical processes such as synaptogenesis, myelination, and the maturation of neuronal circuits [19,20]. During this stage, the transplacental transfer of nutrients, particularly DHA and other essential fatty acids, peaks to meet the increasing nutritional demands of the developing fetus [21,22]. Consequently, the study of nutritional influences, including fatty acid ratios, should consider these gestational periods separately to capture their distinct contributions to neurodevelopment.

In summary, the evidence regarding the relationship between maternal circulating *n*-6/*n*-3 ratios and offspring neurodevelopment is limited and inconclusive. Therefore, to contribute to the existing knowledge, we aim to analyze the association between maternal serum concentrations of *n*-6/*n*-3 ratios in the first and third trimesters of pregnancy and the neurodevelopment of their children in the early days, in a community sample from a Mediterranean region of northern Spain.

## 2. Materials and Methods

### 2.1. Study Design and Procedure

This is a prospective follow-up study of pregnant women from their first trimester of pregnancy up until the postpartum period taking part in the ECLIPSES cohort study, a community randomized controlled trial (RCT) conducted in a Mediterranean area from the north of Spain [23,24]. A total of 791 participants were recruited from primary care centers before the 12th week of gestation and were followed up at the 12th, 24th, and 36th weeks of gestation and at early postpartum (6–8 weeks postpartum). The inclusion criteria were to be more than 18 years old, to have a pregnancy of less than 12 weeks of gestation, and to understand Spanish or Catalan. The exclusion criteria included multiple pregnancies, to have anemia (Hb < 110 g/L) at the beginning of the study, to have hypersensitivity to egg protein, and to have previous severe illnesses (immunosuppression, malabsorption syndromes, diabetes, cancer, and hepatopathies).

Maternal blood samples, sociodemographic, clinical, and lifestyle habits, as well as infant neurodevelopment information were collected.

**Figure 1:**
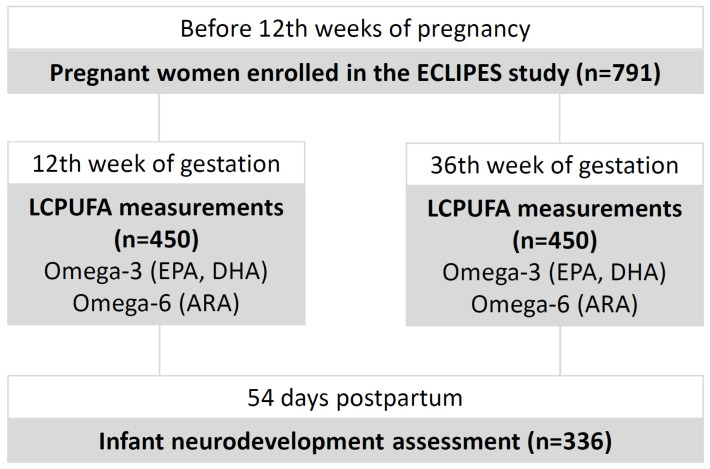
Flow chart of the study design, measurements, and population.

LCPUFA serum concentrations were determined at 12 and 36 weeks of gestation in a subsample of 450 participants, 336 of whom attended the early postpartum infant cognitive assessment follow-up visit. In Figure 1 there is the flow chart of this study.

The ECLIPSES study was approved by the ethics committee of the Pere Virgili Institute for Health Research (IISPV) (155/2017) and by the Spanish Agency for Medicines and Medical Devices (AEMPS). Informed consent was signed by all the participants of this study. This study adheres to the principles of the Declaration of Helsinki.

### 2.2. Instruments and Data Collection

#### 2.2.1. Main Measurements

Maternal serum concentrations of *n*-3 LCPUFA (e.g., EPA and DHA) (μmol/L) and *n*-6 LCPUFA (e.g., ARA) (μmol/L) were determined using LC-MS/MS. Serum samples were collected after a period of fasting. The serum was centrifuged and stored at −80 °C until analysis. A standard internal mixture was combined with 20 μl of serum in methanol to precipitate proteins. The supernatant was mixed with water, O-Benzylhydroxylamine (BHA, Sigma-Aldrich, St. Louis, MO, USA), and *N*-(3 Dimethylaminopropyl)-N0-ethyl carbodiimide (EDC, Sigma-Aldrich) to obtain PUFA derivatives. PUFA derivatives were purified by liquid–liquid extraction using diethyl ether and by ultra-performance liquid chromatography-mass spectrometry (UHLC-MS/MS) using a UHPLC 1290 Infinity II Series coupled to a QqQ 6470 Series^®^ (Agilent Technologies Inc., Santa Clara, CA, USA) and analyzed. Chromatographic separation was performed by gradient elution using a ternary mobile phase containing water, methanol, and isopropanol in ammonium format on a Kinetex Polar C18 analytical column (2.6 μm, 2.1 × 100 mm) (Phenomenex, Torrance, CA, USA). The mass spectrometer was operated in multiple reaction monitoring (MRM) modes, and PUFAs were ionized by positive electrospray. The UHPLC-MS/MS system was monitored by an Agilent MassHunter^®^ workstation (Agilent Technologies Inc., Santa Clara, CA, USA). Samples were analyzed in duplicate, and the average of the two values was computed. The total *n*-3 PUFA concentration was calculated as the sum of EPA and DHA, while the total *n*-6 PUFA concentration included ARA. The *n*-6/*n*-3 ratio was calculated by dividing the total *n*-6 PUFA concentration by the total *n*-3 PUFA concentration.

The neurodevelopment of infants was assessed in the follow-up postpartum visit, approximately 40 days after delivery, using the third edition of the Bayley Scales for Infant and Toddler Development (BSID-III) [25], an individual examination that assesses the developmental performance of infants aged from 0 to 42 months. The BSID-III comprises three general scales (cognitive scale, language scale, and motor scale) and four subscales (expressive language, receptive language, fine motor, and gross motor). In around 1.5–2 months old infants, the cognitive scale evaluates sensory–motor development, visual attention, and exploration; the language scale, which includes the receptive and expressive language subscales, assesses the infant’s receptive communication, preverbal behavioral abilities, sound differentiation, and social and object orientation; and the motor scale, which includes the fine and gross motor scales, assesses prehension, perceptual–motor integration, visual object tracking, response to tactile information, limb and torso movement, static positioning, and balance. The BSID-III was administered by two trained psychologists during a visit around 6–8 weeks postpartum, with parents or main caregivers present.

#### 2.2.2. Adjustment Measurements

Socioeconomic Status (SES) was determined using the Hollingshead index [26], by combining data on the education and job of the mother and the couple (if there is one), and three categories were obtained (high, medium, and low).

Pregnancy smoking status was assessed using the Fagerström questionnaire (Fagerström_Q) [27], and women were categorized into two groups: smokers and non-smokers.

Physical activity was measured at the 12th and 36th weeks of gestation by the short version of the International Physical Activity Questionnaire (IPAQ-SF) [28] which asks for the type, frequency, and duration of physical activity performed in a typical week to obtain a metabolic equivalent waste (MET) per minute per week (MET-min/week).

The mother’s weight (kg) and height (cm) were measured to calculate her Body Mass Index (BMI) at the 12th and 36th weeks of gestation, and differences in BMI were computed to determine BMI changes in pregnancy.

Serum short-chain fatty acids (SCFAs) (acetic acid, propionic acid, and butyric acid) were measured using liquid chromatography–tandem mass spectrometry (LC-MS/MS).

The serum ferritin (μg/L) level was determined by immunochemiluminescence.

The serum B12 vitamin (µmol/L) levels were determined using a chemiluminescent immunoassay on an ADVIA Centaur analyzer using a commercial kit (ADVIA Centaur IRI, Siemens Healthcare Diagnostics Inc., Tarrytown, NY, USA).

Data for obstetric and neonatal variables were collected from each woman’s obstetric clinical report. These variables included the infant’s sex, neonatal birth weight (measured with SECA electronic scales accurate to 10 g), and gestational age at birth (verified by ultrasound in obstetric examinations).

Infant feeding (breastfeeding or formula feeding) was recorded at the moment of developmental assessment.

### 2.3. Statistical Analysis

The data were analyzed using the SPSS statistical software version 29.0.

The normality of the variables was assessed using the Kolmogorov–Smirnov test. Descriptive statistics for quantitative variables were presented as means and standard deviations (SDs) or medians (interquartile range, IQR), whereas categorical variables were presented as the number of observations and the percentage (%). Comparisons between variables were performed using the Student’s *t*-test for parametric variables and the Wilcoxon signed-rank test for non-parametric variables.

The *n*-6/*n*-3, ARA/DHA, and ARA/EPA ratios were computed. The ratios were divided into two categories based on the 75th percentile. The <75th-percentile category was defined as the reference.

Multiple linear regression analyses were performed to examine the relationship between each measurement of neonatal neurodevelopment in the early days as a continuous outcome (cognitive, language, receptive language, expressive language, motor, fine motor, and gross motor) and each of the ratios of maternal essential fatty acids in both the first and third trimesters separately as a categorical variable (according to the 75th percentile) and as a quantitative variable. The unadjusted and adjusted models were computed. The adjustment variables considered were family socioeconomic status (high, medium, or low), tobacco consumption (yes or no); BMI change (Kg/m^2^); physical activity (MET-min/week); vitamin B12 (µmol/L); serum ferritin (µg/L); acetic acid (µmol/L); propionic acid (µmol/L); butyric acid (µmol/L); iron supplementation in the RCT (20, 40, and 80 mg/d); gestational age at birth (weeks); infant’s sex (boy or girl); and type of feeding (formula or breastfeeding). Levels *p* < 0.05 were considered statistically significant.

## 3. Results

### 3.1. Descriptive Characteristics of the Sample

The descriptive characteristics of the participants are shown in Table 1.

This study included a total of 336 pregnant women with a mean age of 38.9 years (SD = 5.0). The socioeconomic status of the families was distributed as follows: 41.1% were in the low socioeconomic category, 43.8% were in the mid category, and 15.2% were in the high category. Regarding lifestyle habits, 85.1% of the women reported no tobacco consumption during pregnancy, while 14.9% did smoke. The newborns had an average gestational age at birth of 39.8 weeks (SD = 1.2) and were evaluated at 47 days (SD = 13) after birth. The sex distribution of the infants was relatively balanced, with 52.7% boys and 47.3% girls.

### 3.2. Multivariate Linear Regression Models for the Associations Between Maternal n-6/n-3, ARA/DHA, and ARA/EPA Ratios and Infant Developmental Domains

The results of the multivariate unadjusted and adjusted linear regression models for the associations between maternal serum concentrations of *n*-6/*n*-3, ARA/DHA, and ARA/EPA ratios in the first and third trimester of pregnancy and infant neurodevelopmental domains are shown in Table 2, Table 3 and Table 4.

In relation to the first-trimester maternal serum *n*-6/*n*-3 ratio (Table 2), ARA/DHA ratio (Table 3), and ARA/EPA ratio (Table 4) concentrations, no significant associations with infant neurodevelopmental domains were identified, neither when the ratios were considered continuously nor when categorized by the 75th percentile.

When the ratios in the third trimester were considered, a significant negative relationship was observed between the maternal serum *n*-6/*n*-3 ratio considered continuously (β = −0.124, *p* = 0.023) and as categorized by the 75th percentile (β = −2.963, *p* = 0.035) and the infant total motor scale (Table 2). In relation to the serum ARA/DHA ratio concentration, a negative association was also observed with infant total motor and fine motor scales both when ratios are considered continuously (β = −2.005, *p* = 0.002 and β = −0.389, *p* = 0.001, respectively) and when categorized by the 75th percentile (β = −3.736, *p* = 0.009 and β = −0.625, *p* = 0.013, respectively) (Table 3).

No significant associations were observed between the maternal serum ARA/EPA ratio concentrations in the third trimester and infant developmental scale outcomes (Table 4).

## 4. Discussion

This cohort study, based on a community sample of pregnant women from a developed country, contributes to the limited evidence on the association of maternal serum levels of *n*-6 and *n*-3 LCPUFAs during gestation on infant neurodevelopment. We observed an association between higher ratios of *n*-6/*n*-3 and ARA/DHA in the third trimester of pregnancy with poorer motor development in infants in the early days postpartum. No such association was found for these ratios in the first trimester nor for the ARA/EPA ratio in the third trimester. This emphasizes the potential role of late gestational fatty acid status in influencing early neurodevelopmental outcomes.

The findings of our study are partly consistent with previous research. Concerning the evaluation of the effect of the *n*-6/*n*-3 ratio in maternal serum levels during pregnancy on infant neurodevelopment, to our knowledge, this has only been studied in the Seychelles Child Development Nutrition Study [16], conducted on 225 mother–baby pairs. They evaluated the *n*-6/*n*-3 ratio in the third trimester of pregnancy and infant neurodevelopment at 9 and 30 months of age, observing that the ratio was associated with poorer psychomotor development at 9 months but not at 30 months. There are some studies that have evaluated this ratio during pregnancy through maternal diet, such as the Maternal and Infant Environmental Health (MOCEH) study conducted with 960 participants in Seoul. This study also observed a negative association between the dietary *n*-6/*n*-3 ratio during pregnancy and mental and psychomotor development at 6 months of age, also assessed using the second edition of the BSID [29]. In school-aged children, the results remain inconclusive, with some studies observing a direct relationship between a higher *n*-6/*n*-3 ratio and emotional [12] and attention problems [14], while studies assessing cognitive function do not find any relationship [15]. The variability in results across studies could be attributed to differences in the gestational timing of fatty acid determination, the age at which child neurodevelopment was assessed, and/or the specific neurodevelopmental domain evaluated, as well as adjustments for potential confounding factors in the analysis. Most studies in this field have conducted a single determination of maternal PUFA levels, typically at the end of gestation [16,30]. This predominance of third-trimester assessments is aligned with the evidence indicating that during this period, there is a substantial increase in the transplacental transfer of fatty acids, particularly DHA, to the growing fetus. This surge likely supports the rapid synaptogenesis, myelination, and other neurodevelopmental milestones occurring during the late fetal stage [3,31]. Our results support this idea since a higher *n*-6/*n*-3 ratio was associated with poorer psychomotor development in the third trimester, whereas no significant association was found in the first trimester. This finding suggests that early neurodevelopmental trajectories may be particularly sensitive to the fatty acid environment during periods of intense brain growth and structural reorganization.

Our results can be explained in several ways. Although some studies have observed beneficial effects of *n*-3 LCPUFAs on brain morphology and function in children and adults [32], there is limited research on the effect of maternal *n*-6/*n*-3 LCPUFA ratio concentrations during gestation on early neurodevelopment. Experimental evidence from in vitro studies, such as Dec et al., indicates that an elevated *n*-6/*n*-3 ratio may disrupt early neuronal differentiation and synaptic activity, likely through pro-inflammatory mechanisms [33]. Furthermore, *n*-6 fatty acids, such as ARA, and *n*-3 fatty acids, such as EPA and DHA, are the parent compounds that produce eicosanoids, which are signaling molecules involved in various physiological processes. However, the ratio of *n*-6 to *n*-3 fatty acids is much higher than the optimal ratio of 1:1 to 4:1. When there is an excessive intake of *n*-6 fatty acids and a deficiency of *n*-3 fatty acids, the body produces eicosanoids from *n*-6 fatty acids in larger quantities than those from *n*-3 fatty acids. The predominance of pro-inflammatory eicosanoids derived from *n*-6 fatty acids could amplify inflammatory responses, with downstream effects on processes such as synaptic pruning and neurogenesis. This is particularly concerning given that neuroinflammation has been linked to altered neuronal connectivity and impaired motor and cognitive outcomes in early infancy [34].

Early central nervous system development is an extremely complex process influenced by multiple genetic, biological, metabolic, and environmental factors. Among these factors are maternal lifestyle habits, exposure to environmental toxins, nutritional status, emotional symptoms, medication use, and even maternal microbiota [35]. In our study, we adjusted the regression models considering several of these factors, such as sociodemographic characteristics (family socioeconomic status); clinical aspects (body mass index during pregnancy); nutritional factors (maternal serum ferritin and vitamin B12 levels); lifestyle habits (tobacco consumption and physical activity practice); obstetric outcomes (gestational age at birth, type of delivery, and infant’s sex); indicators of maternal microbiota (acetic, propionic, and butyric acid levels); and infant type of feeding at the time of the developmental assessment. This adjustment allowed us to assess the association between the *n*-6/*n*-3 ratio independently of other effects.

Our results should be interpreted considering the strengths and limitations of our study. One of the key strengths is the early assessment of infant neurodevelopment, which allows us to better isolate the gestational effects of maternal *n*-6/*n*-3 ratios before the impact of postnatal variables could occur. This study was conducted on a large sample of community-based pregnant women, with serum LCPUFA levels determined in both the first and third trimesters of pregnancy, allowing us to determine the actual status regardless of supplementation or diet. Furthermore, infant neurodevelopment was assessed using the third edition of the BSID-III, a tool specifically designed to evaluate early cognitive development and widely used internationally, facilitating the comparison of our results with those obtained by other studies [36,37]. However, the generalization of our results is limited to Mediterranean-ancestry groups with similar social and environmental characteristics to those of Southern Europe, excluding populations with different dietary patterns and genetic backgrounds. Additionally, we acknowledge the relevance of other psychosocial factors, such as parental mental health, family functioning, perceived social support, and attachment, between others, which could interact with prenatal factors and influence neurodevelopmental outcomes in later stages of childhood. These factors should be considered in future studies to better understand the interaction between prenatal and postnatal factors in long-term follow-up research.

## 5. Conclusions

In conclusion, our findings indicate that a higher maternal *n*-6/*n*-3 ratio, estimated in the third trimester of pregnancy, is associated with poorer neurological development in infants within a few days of birth. These results highlight the potential importance of balancing maternal long-chain polyunsaturated fatty acid status to optimize fetal neurodevelopment and contribute to the understanding of potential nutritional influences on early neurodevelopment and may inform future research on dietary recommendations or public health strategies aimed at optimizing maternal and infant health. Further research is required to expand and verify the current state of knowledge.

## Figures and Tables

**Table 1:** Descriptive data of the mother and offspring: sociodemographic, lifestyle, nutrition, and psychological (*n* = 336).

Maternal Characteristics During Pregnancy	First Trimester	Third Trimester	*p*-Value
Age (years), mean ± SD	38.9 ± 5.0		
Family socioeconomic level, *n* (%)			
Low	138 (41.1)		
Mid	147 (43.8)		
High	51 (15.2)		
BMI (kg/m^2^), median (IQR)	23.73 (5.55)	28.02 (6.80)	<0.001
BMI change (kg/m^2^), mean ± SD	4.0 ± 1.4		
Physical activity (MET-min/week), mean ± SD	2325.4 ± 2304.0		
Tobacco consumption, *n* (%)			
No	286 (85.1)		
Yes	50 (14.9)		
*n*-3 (μmol/L), median (IQR)	264.31 (130.14)	248.84 (112.16)	**0.007**
DHA (μmol/L), median (IQR)	234.50 (111.03)	223.77 (94.94)	0.566
EPA (μmol/L), median (IQR)	30.95 (27.85)	18.90 (20.47)	**<0.001**
*n*-6 (μmol/L), median (IQR)	4135.46 (2051.20)	6772.21 (3764.21)	**<0.001**
ARA (μmol/L), median (IQR)	791.72 (371.83)	690.81 (280.75)	**<0.001**
*n*-6/*n*-3 (μmol/L), median (IQR)	16.05 (6.35)	27.62 (14.25)	**<0.001**
ARA/DHA (μmol/L), median (IQR)	3.47 (1.35)	3.13 (1.26)	**<0.001**
ARA/EPA (μmol/L), median (IQR)	25.85 (22.86)	37.09 (33.26)	**<0.001**
Vitamin B12 (µmol/L), median (IQR)	265.68 (109.78)	214.76 (87.45)	**<0.001**
Serum ferritin (µg/L), median (IQR)	33.00 (34.63)	14.50 (11.10)	**<0.001**
Acetic acid (μmol/L), median (IQR)	46.50 (20.89)	44.90 (18.12)	0.756
Propionic acid (μmol/L), median (IQR)	3.50 (1.17)	3.30 (1.03)	0.093
Butyric acid (μmol/L), median (IQR)	0.73 (0.40)	0.80 (0.46)	**<0.001**
Obstetrical outcomes			
Gestational age at birth (weeks), mean ± SD	39.8 ± 1.2		
Baby characteristics			
Infant’s age (days)	47 ± 13		
Infant’s sex, *n* (%)			
Boy	177 (52.7)		
Girl	159 (47.3)		
Type of feeding, *n* (%)			
Formula	61 (18.2)		
Breastfeeding	275 (81.8)		
Infant cognitive development, mean ± SD			
Cognitive Scale	102.0 ± 8.1		
Language Scale	96.4 ± 8.2		
Receptive	10.6 ± 2.0		
Expressive	8.1 ± 1.5		
Motor Scale	108.0 ± 10.4		
Fine	11.5 ± 1.9		
Gross	11.1 ± 2.3		

Values are expressed as a mean ± SD (standard deviation) or *n* = number (%). Abbreviations: BMI, body mass index; *n*-6, omega-6; *n*-3, omega-3; DHA, docosahexaenoic acid; ARA, arachidonic acid; EPA, eicosapentaenoic acid; IQR, interquartile range. The *p*-value for the differences across first trimester vs. third trimester as derived from the Wilcoxon signed-rank test, as appropriate. The significance of the numbers in bold is a *p*-value < 0.05.

**Table 2:** Multivariate-adjusted linear regression models for the associations between the maternal serum *n*-6/*n*-3 ratio in the first and third trimesters and the neurodevelopment of infants.

		T1			T3		
		β	CI	*p*	β	CI	*p*
		Cognitive					
Continuous							
	Unadjusted model	0.058	−0.116, 0.232	0.512	−0.036	−0.116, 0.043	0.367
	Adjusted model	0.102	−0.094, 0.298	0.306	−0.011	−0.094, 0.073	0.802
High (≥75th Pctl)							
	Unadjusted model	0.241	−1.789, 2.271	0.816	−0.525	−2.601, 1.551	0.619
	Adjusted model	1.010	−1.202, 3.223	0.370	0.013	−2.133, 2.158	0.991
		Language scale					
Continuous							
	Unadjusted model	0.033	−0.142, 0.207	0.713	−0.041	−0.121, 0.040	0.319
	Adjusted model	0.126	−0.074, 0.327	0.217	−0.019	−0.104, 0.067	0.671
High (≥75th Pctl)							
	Unadjusted model	0.466	−1.574, 2.506	0.654	−1.274	−3.377, 0.829	0.234
	Adjusted model	1.426	−0.842, 3.693	0.217	−0.939	−3.135, 1.256	0.400
		Receptive language					
Continuous							
	Unadjusted model	0.029	−0.015, 0.074	0.198	0.009	−0.011, 0.029	0.358
	Adjusted model	0.036	−0.016, 0.087	0.174	0.009	−0.013, 0.032	0.398
High (≥75th Pctl)							
	Unadjusted model	0.297	−0.224, 0.819	0.263	0.059	−0.475, 0.593	0.827
	Adjusted model	0.378	−0.204, 0.960	0.202	0.014	−0.552, 0.581	0.960
		Expressive language					
Continuous							
	Unadjusted model	−0.018	−0.051, 0.015	0.278	−0.023	−0.038, −0.008	**0.003**
	Adjusted model	0.007	−0.030, 0.045	0.698	−0.016	−0.032, 0.000	0.052
High (≥75th Pctl)							
	Unadjusted model	−0.141	−0.525, 0.244	0.472	−0.498	−0.897, −0.099	**0.015**
	Adjusted model	0.110	−0.311, 0.531	0.606	−0.343	−0.755, 0.068	0.102
		Motor scale					
Continuous							
	Unadjusted model	−0.039	−0.263, 0.184	0.728	−0.111	−0.213, −0.009	**0.033**
	Adjusted model	−0.084	−0.330, 0.162	0.502	−0.124	−0.232, −0.017	**0.023**
High (≥75th Pctl)							
	Unadjusted model	−0.972	−3.578, 1.690	0.464	−2.778	−5.446, −0.111	**0.041**
	Adjusted model	−1.150	−3.927, 1.628	0.416	−2.963	−5.713, −0.213	**0.035**
		Fine motor					
Continuous							
	Unadjusted model	−0.019	−0.060, 0.022	0.365	−0.021	−0.040, −0.003	**0.026**
	Adjusted model	−0.019	−0.065, 0.027	0.421	−0.018	−0.038, 0.002	0.072
High (≥75th Pctl)							
	Unadjusted model	−0.426	−0.904, 0.053	0.081	−0.499	−0.988, −0.009	**0.046**
	Adjusted model	−0.403	−0.925, 0.118	0.129	−0.473	−0.943, 0.069	0.090
		Gross motor					
Continuous							
	Unadjusted model	0.014	−0.035, 0.063	0.573	−0.015	−0.038, 0.007	0.183
	Adjusted model	0.000	−0.055, 0.054	0.990	−0.023	−0.047, 0.001	0.058
High (≥75th Pctl)							
	Unadjusted model	0.145	−0.430, 0.719	0.621	−0.373	−0.960, 0.214	0.212
	Adjusted model	0.055	−0.558, 0.667	0.861	−0.506	−1.113, 0.101	0.102

Linear regression models were used to calculate the β coefficient (β) and 95% confidence interval (95% CI). Adjusted model for family socioeconomic level (high, medium, and low); tobacco consumption (yes, no); BMI change (Kg/m^2^), body mass index; physical activity (MET-min/week); vitamin B12 in the first or third trimesters (µmol/L); serum ferritin (μg/L) in the first or third trimesters; acetic acid (μmol/L) in the first or third trimesters; propionic acid (μmol/L) in the first or third trimesters; butyric acid (μmol/L) in the first or third trimesters; clinical trial supplementation: 20, 40, and 80 (mg/d); gestational age at birth (weeks); infant’s sex (boy, girl); type of feeding (formula, breastfeeding). The significance of the numbers in bold is *p*-value < 0.05. Abbreviation: *n*-6, omega-6; *n*-3, omega-3.

**Table 3:** Multivariate-adjusted linear regression models for the associations between the maternal serum ARA/DHA ratio in the first and third trimesters and the neurodevelopment of infants.

		T1			T3		
		β	CI	*p*	β	CI	*p*
		Cognitive					
Continuous							
	Unadjusted model	−0.253	−1.189, 0.682	0.595	−0.449	−1.387, 0.488	0.346
	Adjusted model	−0.103	−1.092, 0.866	0.838	−0.396	−1.372, 0.580	0.425
High (≥75th Pctl)							
	Unadjusted model	−1.296	−3.325, 0.733	0.210	−1.686	−3.750, 0.377	0.109
	Adjusted model	−0.555	−2.679, 1.568	0.607	−1.565	−3.749, 0.619	0.159
		Language scale					
Continuous							
	Unadjusted model	−0.059	−0.991, 0.872	0.901	0.030	−0.928, 0.988	0.951
	Adjusted model	0.212	−0.792, 1.215	0.679	0.221	−0.786, 1.227	0.666
High (≥75th Pctl)							
	Unadjusted model	0.051	−1.974, 2.075	0.961	0.533	−1.580, 2.646	0.620
	Adjusted model	0.635	−1.522, 2.791	0.563	0.986	−1.268, 3.240	0.390
		Receptive language					
Continuous							
	Unadjusted model	0.075	−0.164, 0.314	0.536	0.025	−0.218, 0.269	0.837
	Adjusted model	0.092	−0.166, 0.351	0.483	0.010	−0.250, 0.270	0.940
High (≥75th Pctl)							
	Unadjusted model	0.147	−0.372, 0.666	0.578	0.259	−0.277, 0.794	0.343
	Adjusted model	0.240	−0.315, 0.796	0.395	0.254	−0.327, 0.836	0.390
		Expressive language					
Continuous							
	Unadjusted model	−0.097	−0.273, 0.080	0.282	−0.016	−0.200, 0.167	0.861
	Adjusted model	−0.021	−0.208, 0.166	0.824	0.062	−0.128, 0.252	0.523
High (≥75th Pctl)							
	Unadjusted model	−0.120	−0.503, 0.264	0.539	−0.067	−0.472, 0.338	0.746
	Adjusted model	−0.012	−0.413, 0.390	0.955	0.087	−0.339, 0.513	0.688
		Motor scale					
Continuous							
	Unadjusted model	−0.128	−1.328, 1.072	0.834	−1.421	−2.620, −0.222	**0.020**
	Adjusted model	−0.203	−1.445, 1.039	0.748	−2.005	−3.241, −0.769	**0.002**
High (≥75th Pctl)							
	Unadjusted model	−1.729	−4.330, 0.872	0.192	−2.573	−5.225, 0.080	0.057
	Adjusted model	−1.369	−4.034, 1.297	0.313	−3.736	−6.521, −0.951	**0.009**
		Fine motor					
Continuous							
	Unadjusted model	−0.173	−0.394, 0.047	0.122	−0.328	−0.548, −0.108	**0.004**
	Adjusted model	−0.153	−0.386, 0.080	0.199	−0.389	−0.617, −0.161	**0.001**
High (≥75th Pctl)							
	Unadjusted model	−0.535	−1.012, −0.059	**0.028**	−0.569	−1.057, −0.081	**0.022**
	Adjusted model	−0.400	−0.900, 0.100	0.116	−0.652	−1.167, −0.137	**0.013**
		Gross motor					
Continuous							
	Unadjusted model	0.132	−0.134, 0.398	0.329	−0.112	−0.377, 0.152	0.403
	Adjusted model	0.085	−0.190, 0.360	0.542	−0.245	−0.520, 0.031	0.081
High (≥75th Pctl)							
	Unadjusted model	−0.040	−0.618, 0.539	0.893	−0.211	−0.794, 0.372	0.477
	Adjusted model	−0.054	−0.645, 0.537	0.858	−0.500	−1.118, 0.119	0.113

Linear regression models were used to calculate the β coefficient (β) and 95% confidence interval (95% CI). Adjusted model for family socioeconomic level (high, medium, low); tobacco consumption (yes, no); BMI change (Kg/m^2^), body mass index; physical activity (MET-min/week); vitamin B12 in the first or third trimesters (µmol/L); serum ferritin (μg/L) in the first or third trimesters; acetic acid (μmol/L) in the first or third trimesters; propionic acid (μmol/L) in the first or third trimesters; butyric acid (μmol/L) in the first or third trimesters; clinical trial supplementation: 20, 40, and 80 (mg/d); gestational age at birth (weeks); infant’s sex (boy, girl); type of feeding (formula, breastfeeding). The significance of the numbers in bold is a *p*-value < 0.05. Abbreviation: ARA, arachidonic acid; DHA, docosahexaenoic acid.

**Table 4:** Multivariate-adjusted linear regression models for the associations between the maternal serum ARA/EPA ratio in the first and third trimesters and the neurodevelopment of infants.

		T1			T3		
		β	CI	*p*	β	CI	*p*
		Cognitive					
Continuous							
	Unadjusted model	0.014	−0.033, 0.060	0.564	0.007	−0.022, 0.035	0.637
	Adjusted model	0.028	−0.020, 0.076	0.257	0.014	−0.016, 0.044	0.350
High (≥75th Pctl)							
	Unadjusted model	0.868	−1.226, 2.963	0.415	0.487	−1.614, 2.588	0.649
	Adjusted model	1.462	−0.681, 3.605	0.180	1.499	−0.693, 3.690	0.179
		Language scale					
Continuous							
	Unadjusted model	0.001	−0.047, 0.048	0.976	0.000	−0.029, 0.028	0.987
	Adjusted model	0.010	−0.041, 0.060	0.706	0.008	−0.022, 0.038	0.605
High (≥75th Pctl)							
	Unadjusted model	−0.485	−2.631, 1.661	0.657	0.185	−1.908, 2.279	0.862
	Adjusted model	−0.439	−2.661, 1.783	0.698	0.727	−1.487, 2.940	0.519
		Receptive language					
Continuous							
	Unadjusted model	0.005	−0.007, 0.017	0.430	0.001	−0.007, 0.008	0.869
	Adjusted model	0.005	−0.007, 0.018	0.407	0.001	−0.007, 0.009	0.832
High (≥75th Pctl)							
	Unadjusted model	0.038	−0.503, 0.580	0.889	0.017	−0.512, 0.546	0.949
	Adjusted model	0.012	−0.557, 0.582	0.966	0.074	−0.494, 0.641	0.799
		Expressive language					
Continuous							
	Unadjusted model	−0.005	−0.014, 0.004	0.297	−0.001	−0.006, 0.005	0.772
	Adjusted model	−0.002	−0.012, 0.007	0.609	0.002	−0.004, 0.007	0.568
High (≥75th Pctl)							
	Unadjusted model	−0.205	−0.616, 0.207	0.328	0.037	−0.365, 0.439	0.857
	Adjusted model	−0.162	−0.571, 0.247	0.436	0.164	−0.254, 0.582	0.441
		Motor scale					
Continuous							
	Unadjusted model	−0.020	−0.079, 0.040	0.520	−0.022	−0.057, 0.013	0.216
	Adjusted model	−0.022	−0.083, 0.039	0.476	−0.031	−0.068, 0.005	0.092
High (≥75th Pctl)							
	Unadjusted model	−0.659	−3.353, 2.035	0.631	−0.975	−3.577, 1.627	0.462
	Adjusted model	−0.817	−3.526, 1.893	0.554	−1.176	−3.888, 1.537	0.394
		Fine motor					
Continuous							
	Unadjusted model	−0.009	−0.020, 0.002	0.098	−0.005	−0.011, 0.002	0.164
	Adjusted model	−0.007	−0.019, 0.004	0.219	−0.005	−0.012, 0.002	0.156
High (≥75th Pctl)							
	Unadjusted model	−0.255	−0.752, 0.243	0.315	−0.182	−0.663, 0.299	0.457
	Adjusted model	−0.178	−0.687, 0.331	0.492	−0.114	−0.617, 0.390	0.658
		Gross motor					
Continuous							
	Unadjusted model	0.002	−0.011, 0.015	0.798	−0.003	−0.011, 0.005	0.435
	Adjusted model	−0.001	−0.015, 0.012	0.853	−0.006	−0.014, 0.002	0.172
High (≥75th Pctl)							
	Unadjusted model	−0.075	−0.669, 0.519	0.804	−0.191	−0.768, 0.387	0.517
	Adjusted model	−0.214	−0.810, 0.381	0.479	−0.309	−0.915, 0.296	0.316

Linear regression models were used to calculate the β coefficient (β) and 95% confidence interval (95% CI). Adjusted model for family socioeconomic level (high, medium, low); tobacco consumption (yes, no); BMI change (Kg/m^2^), body mass index; physical activity (MET-min/week); vitamin B12 in the first or third trimesters (µmol/L); serum ferritin (μg/L) in the first or third trimesters; acetic acid (μmol/L) in the first or third trimesters; propionic acid (μmol/L) in the first or third trimesters; butyric acid (μmol/L) in the first or third trimesters; clinical trial supplementation: 20, 40, and 80 (mg/d); gestational age at birth (weeks); infant’s sex (boy, girl); type of feeding (formula, breastfeeding). The significance of the numbers in bold is a *p*-value < 0.05. Abbreviation: ARA, arachidonic acid; EPA, eicosapentaenoic acid.

## Data Availability

The datasets generated and/or analyzed during the current study are not publicly available due to subject confidentiality but are available from the corresponding author upon reasonable request.
